# Perspective: The Potential Effects of Naringenin in COVID-19

**DOI:** 10.3389/fimmu.2020.570919

**Published:** 2020-09-25

**Authors:** Ricardo Wesley Alberca, Franciane Mouradian Emidio Teixeira, Danielle Rosa Beserra, Emily Araujo de Oliveira, Milena Mary de Souza Andrade, Anna Julia Pietrobon, Maria Notomi Sato

**Affiliations:** ^1^Laboratory of Dermatology and Immunodeficiencies, LIM-56, Department of Dermatology, School of Medicine and Institute of Tropical Medicine of São Paulo, University of São Paulo, São Paulo, Brazil; ^2^Institute of Biomedical Sciences, University of São Paulo, São Paulo, Brazil

**Keywords:** antiviral, anti-inflammatory, naringenin, SARS-CoV-2, COVID-19, TNF, ACE2

## Abstract

Coronavirus disease 2019 (COVID-19), caused by Severe Acute Respiratory Syndrome Coronavirus-2 (SARS-CoV-2), was declared a pandemic by the World Health Organization in March 2020. Severe COVID-19 cases develop severe acute respiratory syndrome, which can result in multiple organ failure, sepsis, and death. The higher risk group includes the elderly and subjects with pre-existing chronic illnesses such as obesity, hypertension, and diabetes. To date, no specific treatment or vaccine is available for COVID-19. Among many compounds, naringenin (NAR) a flavonoid present in citrus fruits has been investigated for antiviral and anti-inflammatory properties like reducing viral replication and cytokine production. In this perspective, we summarize NAR potential anti-inflammatory role in COVID-19 associated risk factors and SARS-CoV-2 infection.

## Introduction

The respiratory diseases named Coronavirus disease 2019 (COVID-19) is generated by a respiratory infection with Severe Acute Respiratory Syndrome Coronavirus-2 (SARS-CoV-2) (1, 2). Due to the rapidl viral transmission, the disease was declared a pandemic by the World Health Organization a few months after the first diagnosed case (3, 4).

Besides the similar clinical manifestations to previous Severe Acute Respiratory Syndrome Coronavirus-1 (SARS-CoV-1), SARS-CoV-2 infection presents a much lower death rate (5). Approximately 5% of patients progress to a severe COVID-19, developing mainly severe acute respiratory syndrome, with 3% needing assisted respiratory mechanic ventilation. Coronavirus disease 2019 can progress to septic shock and multiple organ failure (6) and exhibits a death rate of approximately 2% (7).

The SARS-CoV-2 can infect human cells by entry via the angiotensin-converting enzyme 2 (ACE2) receptor and Transmembrane Serine Protease 2 (TMPRSS2) (8). Although this process is wildly accepted, other possible infective routes are being explored such as antibody-dependent enhancement (ADE) (7) and via CD147 (9).

Angiotensin-converting enzyme 2 expression is one of the main explanations for the higher airway infection, as it is highly expressed in the respiratory tract such as epithelial cells of the alveoli, trachea, and bronchi, some bronchial glands and alveolar macrophages (10). However, ACE2 is also expressed in the ileo, kidney, adipose tissue, heart, brain, blood vessels, stomach, liver, and oral and nasal mucosas (11), which could corroborate the systemic inflammatory profile in COVID-19.

Upon viral entry, the virus induces the host to increase the production and release of inflammatory cytokines, which can lead to greater immune activation and tissue damage (12). Hypothetically, the reduction of inflammation could aid COVID-19 patients (13).

**FIGURE 1:**
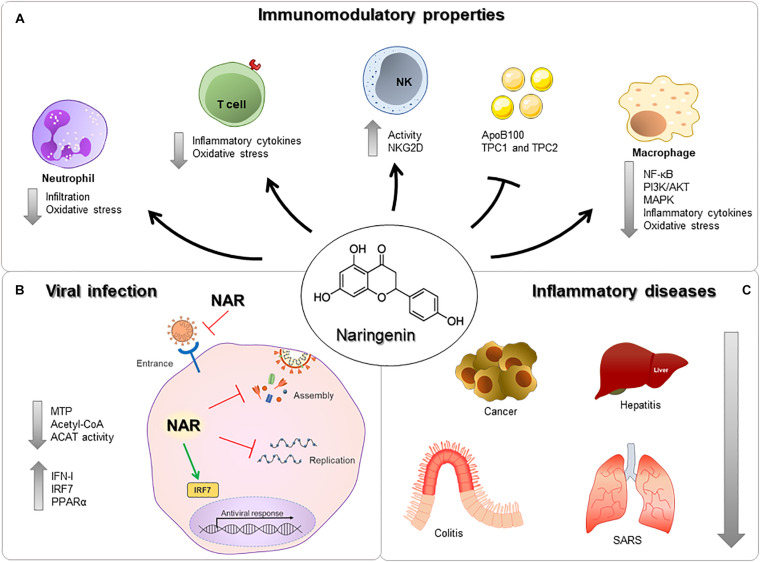
Immunomodulatory properties of nargenin. **(A)** NAR can act on Neutrophils, T cells, NK cells, Macrophages, and reduce the expression of proteins and receptors. **(B)** NAR reduces viral entry, assembly, and replication via modulation of surface molecules, production of antiviral components, inflammatory molecules and/or direct interaction with viral components. **(C)** NAR can influence the development and severity of many different diseases, in different organs, such as cancer, hepatitis, colitis, and severe acute respiratory syndrome.

Several compounds have been associated with antiviral and anti-inflammatory properties and could impact COVID-19 development such as vitamin D (14), vitamin E (15), vitamin B12 (16), omega-3 (17), and flavonoids (18). Naringenin (NAR) is an important natural flavonoid present in citrus fruits, like grapefruit (43.5 mg/100 mL) and oranges (2.13 mg/100 mL) (19), with a high analgesic, anti-oxidant, anti-inflammatory, anti-tumoral, and anti-viral effect (20–23) (Figure 1). The consumption of 8 mL/kg of orange juice increases NAR plasma levels from 0 to 300 μg/L 4 h after ingestion (24).

The antiviral effect of NAR has been studied in several viruses, such as dengue (25, 26), hepatitis C (27), zika (28), chikungunya (29), Semliki Forest (30), herpes simplex 1 and 2 (31), yellow fever (32), and human immunodeficiency virus (33). Several *in vitro* studies have highlighted NAR’s antiviral effect in pre-infection and post-infection (28). Similar to other natural compounds, NAR has extensively been investigated in *in vitro*, but has very limited results in *in vivo* models of viral infection (34, 35) (Figure 1B). Nevertheless, the *in vitro* and *in vivo* anti-inflammatory potential of NAR has been highlighted in several animal models, including respiratory syndromes (35, 36). In this perspective, we highlight the mechanism in which NAR may present an important anti-inflammatory role in COVID-19.

## Anti-Inflammatory Properties of Naringenin

Inflammation can be characterized by the regulation of pro- and anti-inflammatory mediators in resident cells and leukocytes recruited from the blood (37). There are strong pieces of evidence of the role of NAR under inflammatory conditions due to a wide range of mechanisms. The immunomodulatory properties of NAR are associated with the regulation of key signaling pathways, like nuclear factor kappa-light-chain-enhancer of activated B cells (NF-κB) (38), PI3K/AKT (23), and mitogen-activated protein kinases (MAPK) (39) in different cell types (Figure 1A).

**FIGURE 2:**
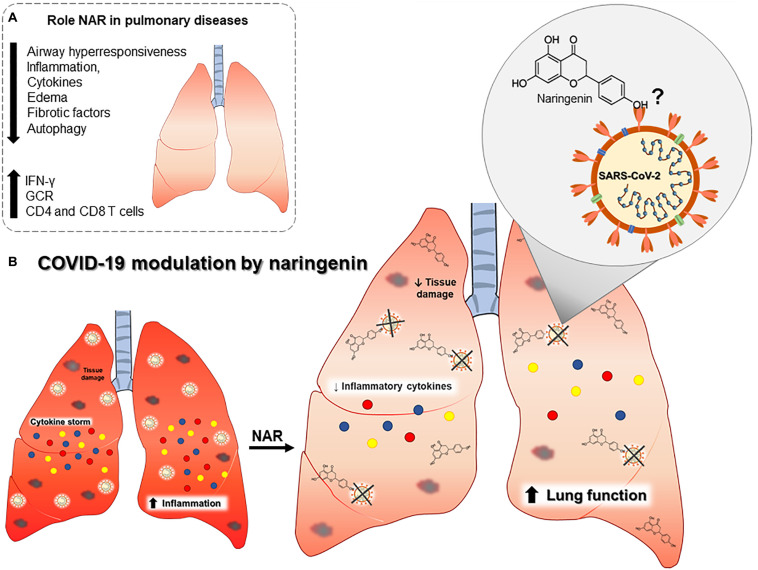
Outline of the putative role of naringenin in COVID-19 pulmonary pathophysiology. **(A)** Established effects of naringenin on different pulmonary diseases. **(B)** Naringenin may reduce inflammatory cytokines and tissue damage, and it may directly bind to SARS-CoV-2. Abbreviations: Interferon gamma (IFN-γ), glucocorticoid receptor expression (GCR), and cluster differentiation (CD).

Macrophages are an important cell in the COVID-19 pathology, being able to sense and respond to pathogens and produce inflammatory cytokines and chemokines (40). In murine macrophages, NAR can reduce inflammatory mediators production induced by LPS, and in a murine endotoxemia model reduces the mortality rates from 60 to 0% (41). Murine macrophages infected with a gram-negative bacteria (*Chlamydia trachomatis*) NAR reduced the production of IL-1β, IL1α, IL-6, TNF, IL-12p70, and IL-10 in a dose-dependent manner (42). Moreover, NAR’s anti-inflammatory effects have been demonstrated *in vivo* (41), in macrophage and *ex vivo* human whole-blood models, reducing IL-1β, IL-6, IL-8, and TNF upon LPS stimulus to close to non-stimulated levels (43) (Figure 2).

Barnes et al. described that the cytokine storm developed by severe COVID-19 patients is related to an exacerbation of neutrophil activation (44). It is clear the central role of neutrophils in COVID-19, as neutrophilia and neutrophil-to-lymphocyte ratio in COVID-19 patients is associated with disease severity (45, 46).

Lung biopsies have also identified an infiltration of neutrophils (47) and the formation of neutrophil extracellular traps in COVID-19 patients (48) (Figure 1A). Although some animals like cats, ferrets, mice, hamsters, and macaques can be infected by SARS-CoV-2, the usage of animal models in COVID-19 is currently limited (49).

In an animal model of acute respiratory distress syndrome (ARDS), a syndrome with an increase in IL-6, TNF, and neutrophils in the lungs, NAR supplementation can reduce neutrophils infiltration and oxidative stress, greatly reducing airway inflammation and lung injury (50). Naringenin reduction of oxidative stress is partially mediated by a curb in the anion superoxide production (51, 52) (Figure 2).

Naringenin can suppress inflammatory molecules production through both transcriptional and post-transcriptional mechanisms (18). In a LPS-induced model of inflammation in a mouse model, NAR suppressed TNF and IL-6 production by macrophages and T lymphocytes without interfering in the toll-like receptor (TLR) cascade but by increasing intracellular cytokine degradation through lysosome-dependent mechanisms (23). These data indicate a potential role in the control of inflammation and oxidative stress-related to airway inflammatory insults (Figure 2A). These anti-inflammatory and anti-oxidant effects are also described in chronic comorbidities like in diabetes mellitus (53, 54), dyslipidemia, hyperinsulinemia, and being overweight (55), which are all risk factors associated with severe COVID-19 (4, 56, 57) (Figure 1C).

In animal experimental models, NAR was able to modulate different inflammation syndromes and at different sites, such as colitis (58), hepatitis (59), obesity (60), cancer (61), and acute respiratory syndrome (36). This is particularly important in COVID-19, because SARS-CoV-2 infection induces a systemic inflammation and can infect many different organs including lungs, heart, liver, brain, kidneys, and the intestines (62).

In addition, NAR can promote lysosome-dependent cytokine protein degradation, which may be important in COVID-19 (63, 64), considering the systemic and cytokine storm during severe COVID-19 (65). In fact, NAR-induced immunomodulation has been demonstrated in airway inflammatory disorders. In a murine asthma model, treatment with NAR reduced airway hyperactivity and airway inflammation, with a reduction in the levels of IL-4 and IL-13 in bronchoalveolar lavage and serum IgE levels as well improvement in lung function assay (66–68). Overall, the treatment with NAR reduced lung eosinophilia to similar levels to non-asthmatic group (66–68).

In lung fibrosis induced by infection with *Mycoplasma pneumoniae*, NAR reduced autophagy-mediated airway inflammation and lung fibrosis (69, 70), and, in a chronic obstructive pulmonary disease (COPD) model, NAR was able to mitigate lung inflammation, reduce the expression of TGF-β, and increase glucocorticoid receptor expression (GCR) (71). Naringenin anti-inflammatory effect was also verified in radiation-induced lung injury, reducing lung inflammation and IL-1β levels (72).

The NAR anti-inflammatory effect is thus not directly mediated to a type 2 or type 1/17 immune response but a regulation of the immune response. Studies have highlighted the increase in T regulatory cells and transforming growth factor-β after NAR consumption via aryl hydrocarbon receptor-mediated pathway (73).

Nevertheless, the excessive regulation of the inflammatory response could impair anti-viral immune response, that has not been previously observed with NAR supplementation. Naringenin can also activate the interferon-stimulated response element and enhance IFN-I production via an increase in the expression of IRF7 (74) and increase NK cell activity via enhanced NKG2D ligand expression (75). Considering the crucial role of NK cells and IFN-I in the anti-viral immune response, NAR may also contribute to the viral load control. Overall, these previous studies demonstrated, *in vivo* and *in vitro*, that NAR is a strong candidate as an adjuvant in reducing airway and systemic inflammation.

## Naringenin and Coronaviruses

Two coronaviruses have been responsible for recent epidemics. In 2002, the SARS-CoV-1 epidemic caused 8,098 cases, with 774 deaths in 11 countries (76–78). In 2012, in the Middle East, another coronavirus also caused Severe Acute Respiratory Syndrome, being named MERS-CoV (79). Until 2020, MERS-CoV had caused 2,494 cases, with 858 associated deaths (77).

Clinical manifestation of SARS-CoV-1 and MERS-CoV is similar. Patients report clinical symptoms such as fever, cough, body pain, headache, and less commonly, diarrhea and nausea (80). However, the need for intensive care and mechanical ventilation is greater in MERS-CoV than in SARS-CoV-1 (81, 82).

Similarly to MERS-CoV and SARS-CoV-1, SARS-CoV-2 infection is mainly transmitted by respiratory droplets expelled from an infected person during sneezes or coughs (83, 84). Severe Acute Respiratory Syndrome Coronavirus-2 surface glycoprotein spike (S protein) binds to ACE2 on the surface of the host’s cell surface. This invading process is the same used by SARS-CoV-1 (85). In comparison, MERS-CoV uses dipeptidyl peptidase 4 (DPP4), a multifunction surface protein to entry into cells (85). Dipeptidyl peptidase 4 is mainly expressed on the kidney, intestine, liver, prostate, and activated leukocytes. Dipeptidyl peptidase 4 is expressed on the lower respiratory tract, glands located in submucosa of the upper respiratory tract, lung macrophages, and alveolar epithelial cells (86).

After these coronaviruses (MERS-CoV, SARS-CoV-1, and SARS-CoV-2) invade the host’s cell, polypeptides are released from the polyproteins by proteolytic processing. The proteolytic process is mediated by papain-like protease (PL^pro^) and 3-chymotrypsin-like protease (3CL^pro^). The 3CL^pro^ cleaves the polyprotein to generate various non-structural proteins, crucial for viral replication (87, 88). Due to the main role of 3CL in coronaviruses viral cycle, inhibitors of 3CL could potentially be used in COVID-19.

Flavonoid inhibition of the 3CL protease has been described in MERS-CoV (89) and SARS (90), but NAR was not among the flavonoids investigated. Nevertheless, in silico analysis demonstrated that NAR has the potential to inhibit SARS-CoV-2 3CL^pro^ (91). A recent study verified that SARS-CoV-1 and SARS-CoV-2 share 99.02% of genetic similarity of 3CL, with only 12 punctual mutations (88), leading to the possible inhibition of 3CL by NAR and other flavonoids.

Another possible mechanism is the inhibition of the two-pore ionic channel (TPC1 and TPC2) (92). Inhibition of TPC1 and TPC2 reduces MERS-CoV infectivity, intracellular traffic (93), and viral replication (93, 94). Due to SARS-CoV-2 viral genome sequencing similarities with MERS-CoV and SARS-CoV-1 (95), it is possible that similar mechanism of inhibition of TPC1 and TPC2 channel be effective in COVID-19, aiding in the reduction of viral replication (96).Interestingly, NAR can inhibit the activity of TPC1 and TPC2 both in humans and plants (97).

NAR is a hydrophilic substance with a higher affinity for the cytoplasmic membrane generating intracellular accumulation of NAR (98). Therefore, this affinity probably enhances intracellular signaling and the modulation of and TPC1 and TPC2 (27). Therefore, the TPC1 and TPC2’s modulation by NAR should be further investigated as a possible anti-coronavirus intervention.

## Discussion

Several reports of natural compounds with anti-SARS-CoV-2 potential are currently being investigated. Substances that may compete with the ACE2 receptor or reduces the ACE2 expression may present an alternative or adjuvant therapy in COVID-19 (99). In fact, NAR consumption has been associated with a reduction in ACE2 expression in the kidneys of rats (100) and could bind directly to the ACE2 receptor (101).

However, nutritional interventions aiming to regulate SARS-CoV-2 entry receptor ACE2 need to be carefully evaluated, as downregulating of ACE2 could also lead to greater inflammation and lung damage (102, 103). Previous reports demonstrated that the oral consumption of NAR can reduce acute lung injury in a mouse model (50) and reduce the production of pro-inflammatory cytokines (18). This is extremely relevant, as a part of COVID-19 lung injury can be classified as ARDS (104).

Coronavirus disease 2019 can also lead to cytokine storm, progress to septic shock, and cause death (105, 106). Modulating the cytokine storm is thus a vital process for treating COVID-19. Naringenin has been used in experimental models to regulate the production of IL-6 and TNF (23), cytokines that are increased in COVID-19 and further increased in severe cases (107, 108). Also in an animal model of septic shock, the consumption of NAR has been demonstrated to reduce kidney damage via an increase in antioxidant enzymes (109).

Studies verified a direct role of NAR in abrogating viral replication in human cells, before (21) and after infection (30). In SARS-CoV2, in silico analysis demonstrated that NAR has the potential to inhibit SARS-CoV-2 3CL^pro^ and consequently inhibit viral replication (91), which still needs to be further verified experimentally.

The consumption of NAR via citrus fruits (110) or supplementation (111) can rapidly increase circulating levels of NAR and increase intracellular levels of NAR (98, 111). An increase in the concentration of NAR in plasma samples can be observed 20 min oral consumption and peaking around 4 h post-consumption (112). In addition, in vitro models have also demonstrated a long-term anti-viral benefit, even after discontinuation of supplementation with NAR (21), although there is little evidence of *in vivo* antiviral activity (35).

Previous clinical trials with the consumption of 500 mL/day for 8 weeks of orange juice, rich in NAR, has demonstrated an adjuvant effect in antiviral therapy (34). The consumption of 340 mL of grapefruit juice per day (containing approximately 210 mg of NAR) also improved cardiac-related measurements in post-menopause women (113). Although NAR is one of the most important naturaly occurring flavonoids, there is a lack of clinical trials and data on pharmacokinetic aspects, metabolic fate, and chemical stability that may limit the usage of this bioactive compound in humans (35).

A caveat of NAR is the oral consumption. Although widely accepted by patients, it could be a barrier in severe COVID-19 patients. Therefore, NAR may be better applied as a prophylactic intervention or on the onset of SARS-CoV-2 infection. The possible effect of NAR on the ACE2 receptor also needs to investigated, as ACE2 reduction could lead to greater inflammation (102, 103). Naringenin is mostly absorbed in the small intestine (114), and differences in microbiota may thus also present an important inter-individual variable (24, 112).

Another caveat is the NAR poor aqueous solubility and bioavailability; currently, the usage of liposomes, nanoparticles, and other formulations may present itself as a solution (115–118).

Furthermore, NAR interactions with the cytochrome P450 (CYP) system need to be evaluated, as NAR can affect drug-metabolizing enzymes and pharmacokinetic of important drugs that may be of regular use or specific in COVID-19 patients (119–121).

In conclusion, NAR potential as an anti-inflammatory nutritional intervention has been demonstrated in many different diseases, such as SARS-CoV-1 and MERS-CoV. Further investigations and clinical trials are needed to help understand the role of NAR consumption in humans during a viral infection, especially in SARS-CoV-2 infection and COVID-19.

## Data Availability Statement

All datasets generated in this study are included in the article/supplementary material.

## Author Contributions

RA: conception, writing, and review. FT: writing, drawing, and review. DB, EO, MA, AP, and MS: writing and review. All authors contributed to the article and approved the submitted version.

## Conflict of Interest

The authors declare that the research was conducted in the absence of any commercial or financial relationships that could be construed as a potential conflict of interest.
